# Voltammetric Quantification of Anti-Cancer Antibiotic Bleomycin Using an Electrochemically Pretreated and Decorated with Lead Nanoparticles Screen-Printed Sensor

**DOI:** 10.3390/ijms24010472

**Published:** 2022-12-28

**Authors:** Jędrzej Kozak, Katarzyna Tyszczuk-Rotko, Radovan Metelka

**Affiliations:** 1 Faculty of Chemistry, Institute of Chemical Sciences, Maria Curie-Skłodowska University, 20-031 Lublin, Poland; 2 Department of Analytical Chemistry, Faculty of Chemical Technology, University of Pardubice, 532 10 Pardubice, Czech Republic

**Keywords:** electrochemically prepared screen-printed carbon electrode, lead nanoparticles, anti-cancer antibiotic, bleomycin, voltammetry, human urine and wastewater sample

## Abstract

In this paper, we report a highly sensitive voltammetric sensor for the determination of the anti-cancer antibiotic bleomycin (BLM) based on a screen-printed carbon sensor that is electrochemically pretreated and decorated with lead nanoparticles in the sample solution (pSPCE/PbNPs). These sensor surface manipulations contribute to significant amplification of the analytical signal and improvement of its shape and repeatability. The effect of the electrochemical behavior of BLM on the pSPCE/PbNPs was examined by electrochemical strategies. CV, EIS, and XPS were used to compare the sensor surface modifications. The effects of the type and pH of the supporting electrolyte and the procedure parameters were optimized. The features of the proposed procedure include: (a) very low limits of detection and quantification (2.8 × 10^−11^ and 9.3 × 10^−11^ M, respectively), (b) linear ranges (1.0 × 10^−10^–2.0 × 10^−9^ M and 2.0 × 10^−9^–2.0 × 10^−8^ M, and (c) a high sensitivity of 0.32 µA/nM. The electrochemical sensor was successfully applied for the determination of BLM in wastewater and reference material of human urine samples.

## 1. Introduction

Cancer is one of the most dangerous diseases and one of the greatest challenges of modern medicine. The use of anti-cancer drugs is still the basic method of treatment [1]. One of the anti-cancer drugs is bleomycin (BLM), which is a mixture of natural structurally related glycopeptide antibiotics produced by the bacterium *Streptomyces verticillus*. Clinically used bleomycin contains mainly bleomycin A2 and B2 and small amounts of other subfractions [2,3,4]. In combination with other chemotherapeutic agents, BLM is used in the treatment of many types of cancer. It is the first-line drug in the treatment of Hodgkin’s lymphoma [5], but it is also used in the treatment of non-Hodgkin lymphomas and head, neck, and skin cancers. In combination therapy with cisplatin and etoposide, it is highly effective against testicular cancer [6]. BLM is also used in the treatment of malignant pleural effusion [7] and in sclerotherapy in patients with vascular malformations [8]. The wide use of BLM results from the fact that it causes myelosuppression and immunosuppression at a relatively low level [7,9].

The anti-tumor activity of BLM is related to the fact that it induces selective DNA cleavage. BLM produces this effect on both single-stranded and double-stranded DNA. In the presence of oxygen, BLM forms binary Fe(II)•BLM complexes with Fe(II) ions. When Fe(II) is oxidized to Fe(III), oxygen is reduced to free radicals, which then induce DNA cleavage, ultimately leading to cell death [7,10,11]. Despite the aforementioned relatively low toxicity of BLM, the use of this substance is, however, associated with serious dose-limiting side effects, such as kidney and lung toxicity. Treatment with BLM may result in pneumonia progressing to irreversible pulmonary fibrosis with high mortality. An important risk factor is the cumulative BLM dose exceeding 300 mg [5,8,11]. Therefore, in order to achieve the best possible results of BLM therapy while minimizing its side effects, it is necessary to develop sensitive and selective methods of BLM determination in clinical samples.

Several methods allowing the determination of BLM can be found in the literature. High-performance liquid chromatography (HPLC) [2,12,13], high-performance liquid chromatography quadrupole-time of flight mass spectrometry (HPLC-QTOF-MS) [4], electrogenerated chemiluminescence (ECL) [7] or radioimmunoassay (RIA) can be mentioned here [9]. However, these methods require expensive equipment, are often time-consuming and labor-intensive, and generate a high consumption of reagents.

As an alternative, electrochemical methods can be proposed; they require relatively inexpensive devices, are simple, very sensitive, and selective, and also allow analyses to be performed in a short time. Only a few papers describing the voltammetric procedures for BLM determination are available. One of them [14] shows the use of a hanging mercury drop electrode (HMDE). The remaining articles [1,11,15] present voltammetric assays based on BLM-induced DNA strand scission. The lowest limit of detection (LOD), 7.4 × 10^−13^ mol L^−1^, was obtained using the procedure described in [1]. Nonetheless, this procedure, similar to those described in [11,15], requires the complicated and time-consuming preparation of a working electrode modifier, which is DNA in this case, and the subsequent immobilization of DNA on the surface of the electrode. The DNA cleavage reaction itself, which is the basis for obtaining the BLM analytical signal, also takes a relatively long time (10 min, while in the case of other works, even several hours).

To the best of our knowledge, there have been no attempts to use screen-printed electrodes (SPEs) in BLM analysis so far. SPEs have been an increasingly popular type of electrode in recent years. They are characterized by low production costs and high commercial availability. The diversity of electrode materials used to produce SPEs and the ease of modification of the electrode surface make them a very versatile tool for the determination of a whole range of substances [16,17,18]. One of the ways to modify the electrode surface is the electrochemical deposition of metal particles, e.g., lead. The lead film electrode exhibited interesting characteristics, such as lower toxicity and volatility compared with the mercury electrodes, a wide potential window, the ability to operate in a wide range of pH media, good reproducibility, simple preparation, and a simple way of electrochemical surface renewal [19]. This paper presents for the first time the use of modified screen-printed electrodes for BLM detection. The use of the electrochemically prepared screen-printed carbon electrode decorated with lead nanoparticles (pSPCE/PbNPs) allowed highly sensitive and selective determination of BLM in urine and also, in environmental samples, in this case, municipal wastewater.

## 2. Results and Discussion

### 2.1. Sensor Characterization

**Figure 1:**
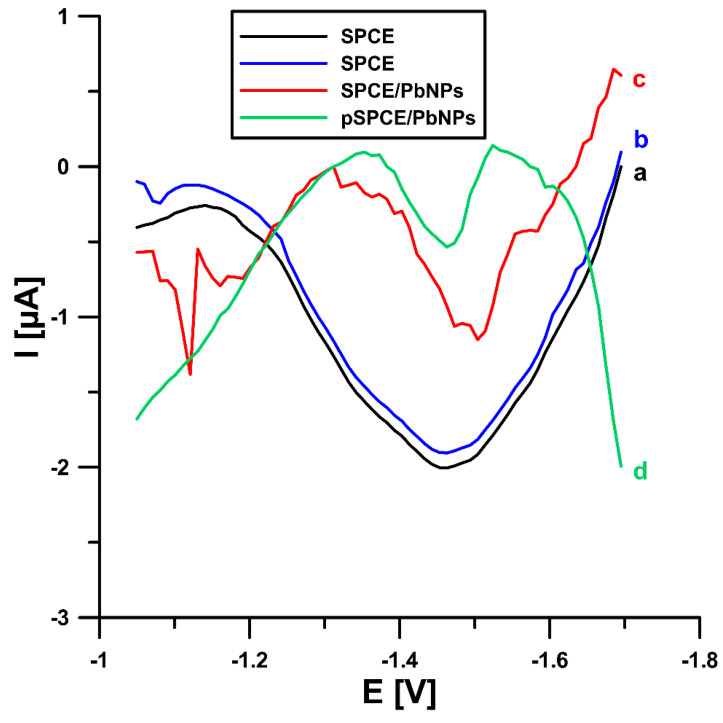
Comparison of the SWAdSV response for: (a and b) unmodified SPCE, (c) SPCE/PbNPs, and (d) pSPCE/PbNPs. BLM concentration of 2 (a, c, and d) and 5 (b) nM. E_acc._ = −1.0 V, t_acc._ = 120 s, f = 50 Hz, E_SW_ = 50 mV, and ΔE = 10 mV.

Initially, the BLM voltammetric response at the electrochemically prepared screen-printed carbon electrode decorated with lead nanoparticles (pSPCE/PbNPs) was examined using square-wave adsorptive stripping voltammetry (SWAdSV). Measurements were made in 0.075 M acetate buffer (pH 4.5) with the addition of 75 µM Pb(II) and 2 nM BLM. Then, under the same conditions, SWAdSV curves were recorded on an unmodified screen-printed carbon electrode (SPCE), and the screen-printed carbon electrode was decorated with lead nanoparticles (SPCE/PbNPs) that had not been pretreated. In Figure 1, it can be seen that the modification of the electrode with PbNPs is necessary to obtain the BLM peak. In the case of the bare SPCE, no BLM reduction signal was observed (2 nM BLM—curve a), which was confirmed by measuring a higher concentration of the analyte (5 nM BLM—curve b). As can be seen, electrochemical pretreatment does not significantly affect the BLM peak current intensity but causes a slight shift of the peak potential towards less negative potential values (−1.50 vs. −1.46 V) and improves the shape of the BLM peak. Moreover, pretreatment of the sensor before its use in a series of BLM measurements significantly improves the repeatability of the signal (5 nM BLM, RSD of 17.74% for the SPCE/PbNPs, and 3.25% for the pSPCE/PbNPs, *n* = 10), which is in line with our previous research [20]. We tried to explain this phenomenon using various analytical techniques.

**Figure 2:**
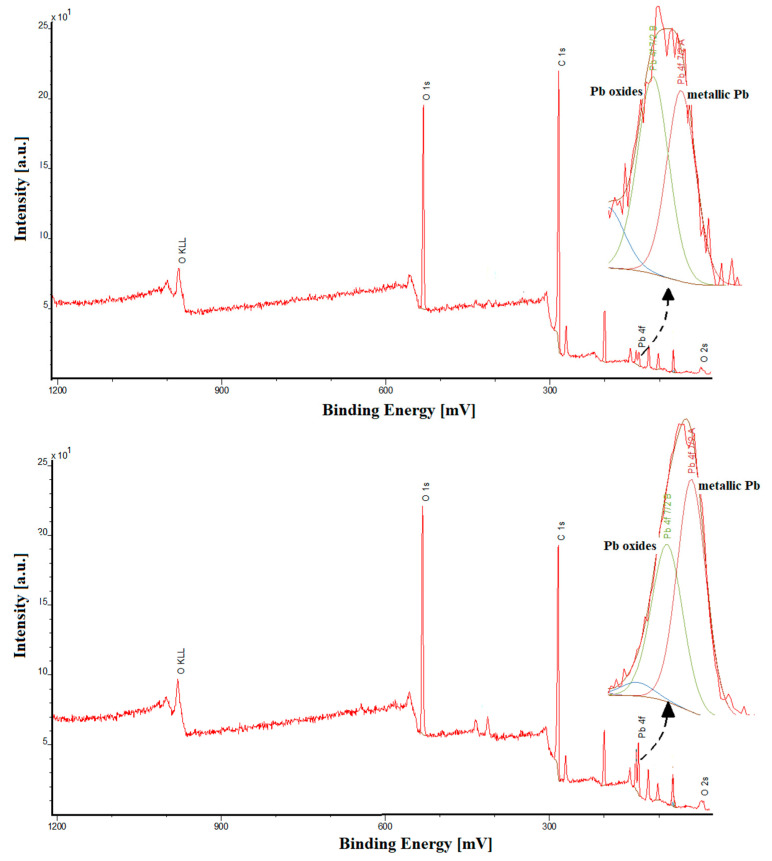
The XPS spectrum of the SPCE/PbNPs and pSPCE/PbNPs, and the deconvoluted Pb4f region.

The pSPCE/PbNPs and the bare SPCE had been characterized using cyclic voltammetry (CV), electrochemical impedance spectroscopy (EIS), scanning and transmission electron microscopy (SEM and TEM), and energy-dispersive X-ray spectroscopy (EDS) in our previous research [20]. In paper [20], we stated that the electrochemically deposited lead nanoparticles were not visible in SEM images, but the PbNPs presence on the electrode surface was confirmed using a TEM–EDS. In this work, the research was supplemented by the analysis of the sensors using X-ray photoelectron spectroscopy (XPS) and the CV and EIS characteristics. The parameters for all tested sensors are summarized in Table 1. The active area of the SPCE/PbNPs electrode was calculated in the same way as for the other electrodes: CV measurements were made in a 0.1 M KCl solution containing 5 mM K_3_[Fe(CN)_6_], and the Randles-Sevcik equation was used here [21]. It can be seen that the active surface area (A_s_) significantly increases with modification with PbNPs (0.072 cm^2^ for the SPCE, 0.23 cm^2^ for the SPCE/PbNPs, 0.22 cm^2^ for the pSPCE/PbNPs). However, electrochemical pretreatment does not contribute to an increase in As. Similarly, in the case of the value of the charge transfer resistance (R_ct_), only the modification with PbNPs causes a slight decrease in the resistance in relation to the unmodified electrode (146.7 Ω cm^2^ for the SPCE, 121.5 Ω cm^2^ for the SPCE/PbNPs, 121.3 Ω cm^2^ for the pSPCE/PbNPs). The XPS analysis shows that the percentage atomic concentration of Pb is as follows: 0.3% for the SPCE/PbNPs and 0.9% for the pSPCE/PbNPs. The higher content of lead is associated with its additional deposition during the electrochemical pretreatment of the sensor. However, this increase does not translate into an increase in As. A very interesting conclusion can be drawn after a careful analysis of the deconvoluted Pb4f signal. The metallic form of lead (55.6%) prevails over lead oxides (i.e., PbO) at the electrochemically pretreated SPCE/PbNPs. In the case of the non-pretreated electrode, an opposite situation is observed since the metallic form of lead accounts for 40.3% of the total deposited lead film. Most probably, the increased proportion of metallic lead affects the repeatability of the deposited lead film before each measurement, thus contributing to the improvement in the shape of the BLM signal and its repeatability. Figure 2 shows the XPS spectrum of the SPCE/PbNPs and pSPCE/PbNPs, and the deconvoluted Pb4f region.

### 2.2. Supporting Electrolyte Composition

**Figure 3:**
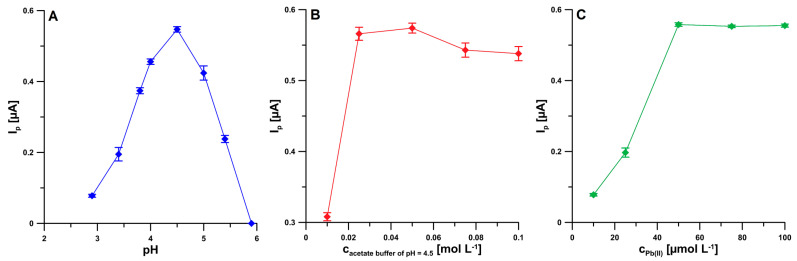
The dependence of pH (**A**), acetate buffer (**B**), and Pb(II) concentration (**C**) on the 2 nM BLM peak current. E_acc._ = −1.0 V, t_acc._ = 120 s, f = 50 Hz, E_SW_ = 50 mV, and ΔE = 10 mV. The received average values of the peak current are shown with a standard deviation for *n* = 3.

The first step in optimizing the procedure was to select the appropriate type and pH of the supporting electrolyte. For this purpose, the influence of the pH value on the reduction signal of 2 nM BLM in the environment of acetic acid and NaAc—HAc solutions with different pH were evaluated. An increase in the BLM peak current was observed with an increase in pH to 4.5, and then a decrease in the BLM signal above this value until it disappeared completely at a pH value of 5.9 (Figure 3A). In view of the obtained results, NaAc—HAc solution (pH 4.5) was selected for further research. Subsequently, the effect of the selected NaAc—HAc buffer concentration on the 2 nM BLM signal was also examined in the range from 0.01 to 0.1 M. The highest peak current was obtained with a buffer concentration of 0.05 M (Figure 3B), and therefore this concentration was considered optimal. The last component of the base electrolyte whose influence on the BLM peak current was checked was Pb(II). The effect of Pb(II) concentration on the 2 nM BLM reduction signal was investigated over a concentration range of 0.01 to 0.1 mM. The highest peak current was obtained at the Pb(II) ion concentration of 0.05 mM (Figure 3C), and this value was chosen for further studies.

### 2.3. Voltammetric Behavior of BLM on the pSPCE/PbNPs

**Figure 4:**
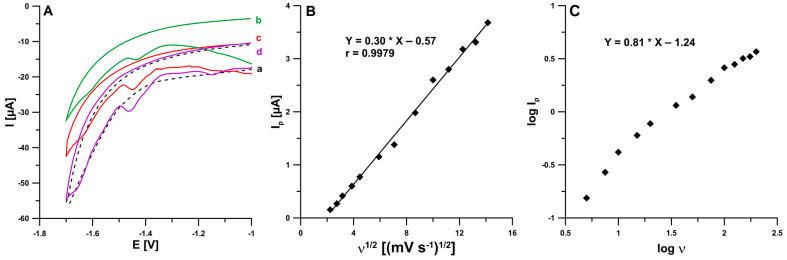
(**A**) CV curves obtained at the pSPCE/PbNPs in the 0.05 M NaAc—HAc buffer (pH 4.5) containing 0.05 mmol L^−1^ Pb(II) and 0 M BLM (dashed line, a) or 0.1 µM BLM (solid line, b, c, and d) (υ = 50, 100 and 200 mV/s). (**B**) The dependence between the BLM peak current (I_p_) and the square root of the scan rate (υ ^1/2^) (υ in the range of 5–200 mV/s). (**C**) The dependence between the log of the BLM peak current (log I_p_) and the log of the scan rate (log υ) (υ in the range of 5–200 mV/s).

The voltammetric behavior of BLM at the pSPCE/PbNPs in the supporting electrolyte solution (0.05 M NaAc—HAc buffer of pH 4.5 and 0.05 mM Pb(II)) containing 0.1 µM BLM was examined using cyclic voltammetry. Figure 4A shows the CVs recorded for the supporting electrolyte (dashed line) and for 0.1 µM BLM (solid line) at a scan rate (υ) of 50, 100, and 200 mV/s. With the potential range used in the measurements, three cathode peaks were visible at potentials of −1.15, −1.45, and −1.65 V (ν = 200 mV/s). However, no oxidation signals were observed, which suggests that the BLM electroreduction process on the electrode is irreversible. Due to the best shape and repeatability of the signal at the potential of −1.45 V on the SWV voltammograms, this peak was selected for further study. In an attempt to determine the nature of the electrode process, the BLM reduction peak current was measured for increasing scan rates ranging from 5 to 200 mV/s. The linear course of the dependence of the peak current (I_p_) on the square root of the scan rate (υ^1/2^) (Figure 4B) suggests that the process is diffusion-controlled, but the slope (0.81) in the plot of the relationship between the log of the peak current and the log of the scan rate (Figure 4C) shows that the process has a mixed nature because it is not fully controlled by diffusion, but also partially by adsorption.

**Figure 5:**
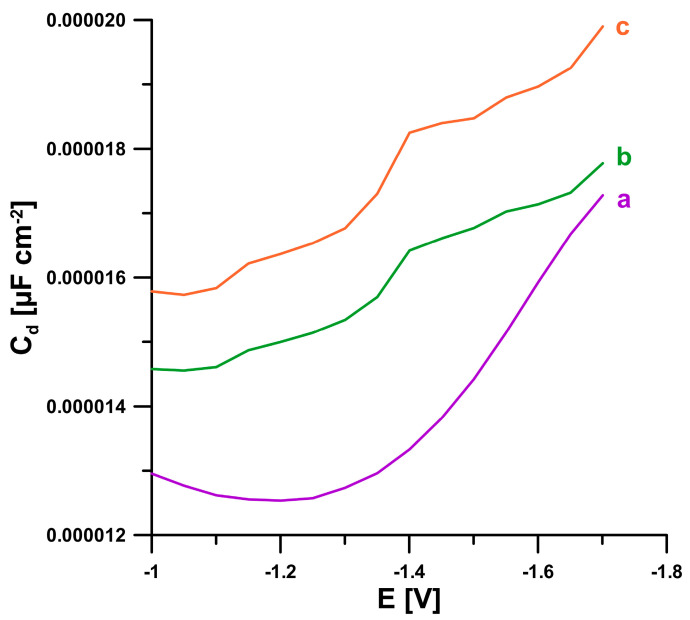
The differential capacity-potential curves of the double layer interface pSPCE/PbNPs/NaAc—HAc buffer (pH 4.5) in the presence of 0 (a), 2 (b), and 5 (c) µM BLM.

The information on the electrochemical response of BLM was also obtained from the analysis of differential capacity curves of the double-layer interface pSPCE/PbNPs/NaAc—HAc buffer (pH 4.5) (frequency of 200 Hz). As shown in Figure 5, in the presence of 2 and 5 µM BLM, three desorption peaks (−1.17, −1.4, and −1.59 V) are visible. This proves the adsorption of BLM onto the electrode surface.

### 2.4. Optimization Step

**Figure 6:**
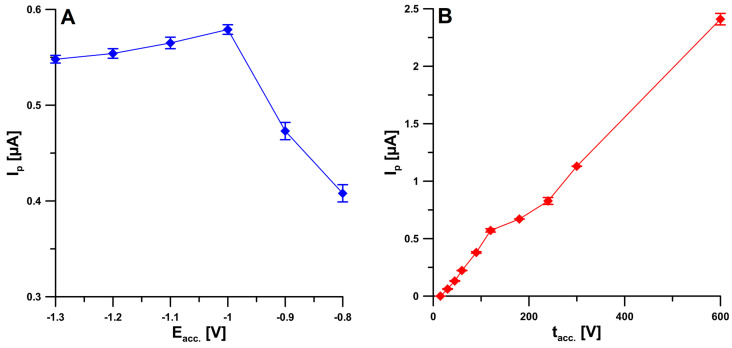
The dependence on E_acc._ (**A**) and t_acc._ (**B**) on the 2 nM BLM peak current. f = 50 Hz, E_SW_ = 50 mV, and ΔE = 10 mV. The received average values of the peak current are shown with a standard deviation for *n* = 3.

In order to achieve the best analytical signal, and thus the best sensitivity and accuracy of the determinations, the SWAdSV procedure parameters were optimized, such as the potential for simultaneous deposition of PbNPs and BLM accumulation on the electrode surface (E_acc._) as well as time (t_acc._), frequency (f), square-wave amplitude (E_SW_), and step potential (ΔE). BLM at a fixed concentration (2 nM) was added to the supporting electrolyte solution, and then the effect of the potential (E_acc._) on the reduction peak ranging from −0.8 to −1.3 V was examined. The highest peak current intensity was obtained at −1.0 V, and therefore this value was considered optimal (Figure 6A). In the next stage, the influence of the time of applying this potential was tested for the selected potential value. A deposition time (t _acc._) of 120 s was chosen; however, since the signal continues to increase with accumulation up to 600 s (Figure 6B), it is possible to attain an even lower detection limit when a longer accumulation time is chosen.

**Figure 7:**
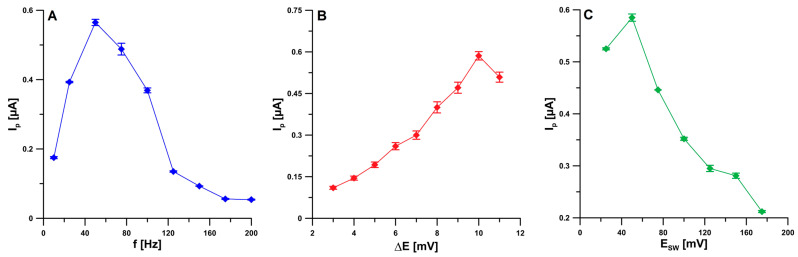
The dependence of f (**A**), ΔE (**B**), and E_SW_ (**C**) on the 2 nM BLM peak current. The received average values of the peak current are shown with a standard deviation for *n* = 3.

For the E_SW_ of 50 mV and the ΔE of 10 mV, the frequency was varied in the range from 10 to 200 Hz. The peak current increased with increasing frequency up to 50 Hz; higher frequencies caused a decrease in the signal, and therefore the value of 50 Hz was selected for subsequent studies (Figure 7A). Then, the influence of ΔE was checked by changing the value of this parameter from 3 to 11 mV. The best result was obtained for the value of 10 mV (Figure 7B). Finally, the effect of E_SW_ values in the range of 25–175 mV was optimized. The highest BLM analytical signal was observed for the E_SW_ of 50 mV (Figure 7C).

### 2.5. Selectivity Studies and Sensor Reproducibility

In order to test the selectivity of the pSPCE/PbNPs sensor, the voltammetric response of 2 nM BLM was checked in the presence of increasing concentrations of potential interferents. It was found that a 2500-fold excess of epinephrine and a 1000-fold excess of Mg(II), Ca(II), glucose, dopamine, ascorbic acid, and uric acid do not significantly alter the peak current of BLM (they do not cause changes greater than 10%). Moreover, a 500-fold excess of V(V), a 200-fold excess of Ni(II), and a 100-fold excess of Fe(III), Cd(II), Cu(II), adenine, and testosterone had negligible effects on the BLM analytical signal. Since natural waters contain surfactants with a surface-active effect corresponding to 0.2–2.0 ppm of Triton X-100 [22], the influence of the presence of 2.0 ppm of this surfactant on the 2 nM BLM signal was also investigated, and no peak current changes exceeding 10% were observed.

Furthermore, three sensors were prepared independently and employed in the SWAdSV analysis of 2 nM BLM. The RSD value equal to 7.5% (*n* = 9) confirms the acceptable reproducibility of the pSPCE/PbNPs sensor.

### 2.6. Voltammetric Determination of BLM

**Figure 8:**
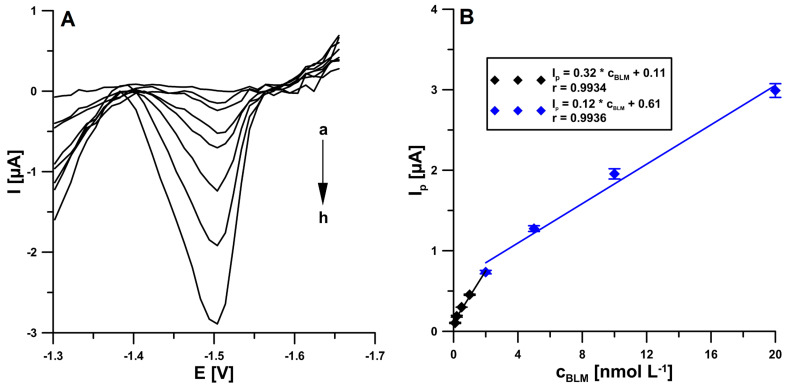
(**A**) The SWAdSV curves obtained on the pSPCE/PbNPs in the presence of increasing BLM concentration (a → h, 0.1–20 nM) in 0.05 M NaAc—HAc buffer (pH 4.5) and 0.05 mM Pb(II). (**B**) Linear ranges of BLM. The received average values of the peak current are shown with a standard deviation for *n* = 3. E_acc._ = −1.0 V, t_acc._ = 120 s, f = 50 Hz, E_SW_ = 50 mV, and ΔE = 10 mV.

The determination of the effect of increasing BLM concentrations on the electrode was performed under optimized conditions using square-wave adsorptive stripping voltammetry (SWAdSV) (Figure 8A). It was observed that the BLM analytical signal increased linearly with increasing concentration over two ranges, the first one from 1 × 10^−^^10^ to 2 × 10^−^^9^ M and the second one from 2 × 10^−^^9^ to 2 × 10^−^^8^ M (Figure 8B). The limits of detection and quantification were calculated to be 2.8 × 10^−^^11^ and 9.3 × 10^−^^11^ M, respectively, using the LOD = 3SD_a_/b and LOQ = 10 SD_a_/b equations (SD_a_—standard deviation of intercept (*n* = 3); b—slope of calibration curve) [23]. The analytical performance of the proposed sensor was compared with other voltammetric BLM determination procedures described in the literature. The collected data are presented in Table 2. Only one of the procedures [1] allows a lower limit of detection to be achieved, but it requires many hours and multi-stage preparation of the working electrode, and the analysis time is relatively long.

### 2.7. Real Samples Analysis

**Figure 9:**
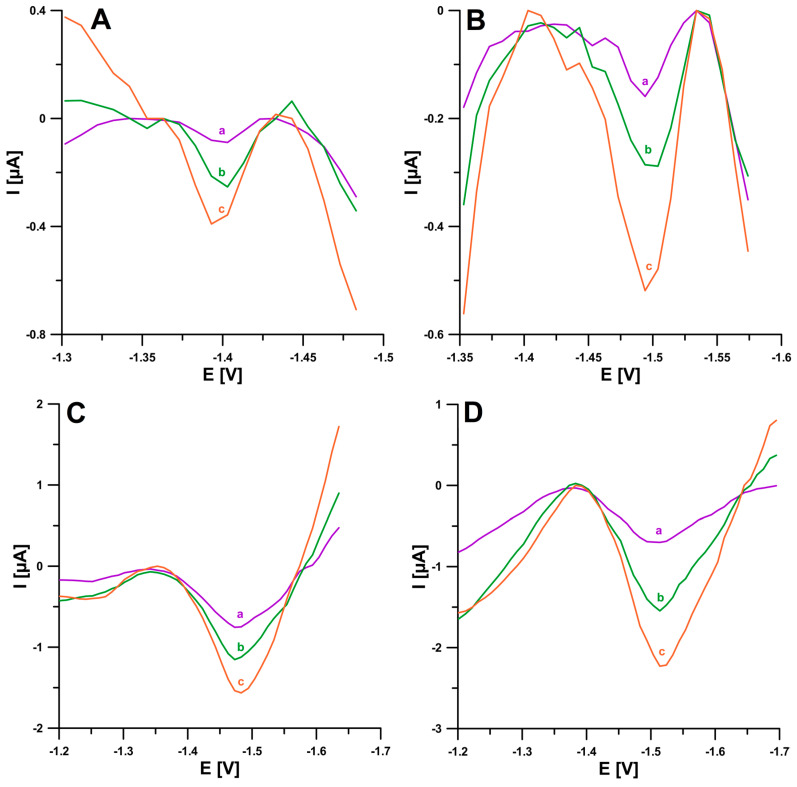
Voltammograms recorded for the determination of BLM in reference material of human urine (**A**,**B**) and wastewater samples purified in a sewage treatment plant (**C**,**D**). (**A**): (a) 1 µL of sample + 2, (b) as (a) + 2, (c) as (a) + 4 nM BLM, (**B**): (a) 1 µL of sample + 4, (b) as (a) + 4 (c) as (a) + 8 nM BLM, (**C**): (a) 1 mL of sample + 0.5, (b) as (a) + 0.5, (c) as (a) + 1 nM BLM, and (**D**): (a) 1 mL of sample + 2, (b) as (a) + 2, (c) as (a) + 4 nM BLM. E_acc._ = −1.0 V, t_acc._ = 120 s, f = 50 Hz, E_SW_ = 50 mV, and ΔE = 10 mV.

The last stage of the research was to confirm the usefulness of the proposed SWAdSV procedure for BLM determination in real samples. Samples of human urine and purified municipal sewage were analyzed. In a single session during BLM therapy, the maximum dose of the drug is 15 IU, which corresponds to 15 g L^−^^1^, and 50–70% of BLM is excreted within 24 h after administration in the urine in the unchanged form [24,25]. Accordingly, the BLM concentrations in the urine of the patients are in the order of 10^−^^5^ M, and therefore, when analyzing human urine samples, multiple sample dilutions (10,000×) could be used. The wastewater samples, on the other hand, were diluted 10× and analyzed with an additional 1 × 10^−^^5^ mol L^−^^1^ DTPA in order to minimize the possible influence of metal ions present in the sample. Spiked samples were analyzed using the standard addition method. The small values of the coefficient of variation (1.16–2.5%) and the values of recoveries (96.0–103.5%) prove the good repeatability of the analytical signal and the good accuracy of the applied method, respectively (Table 3). Figure 9 shows the voltammograms obtained during the determination of BLM in the human urine and municipal sewage samples.

## 3. Materials and Methods

### 3.1. Apparatus

The electrochemical studies were performed using a µAutolab electrochemical analyzer (Eco Chemie, Utrecht, Netherlands) controlled by GPES 4.9 software (voltammetric measurements) and FRA 4.9 software (electrochemical impedance spectroscopy (EIS) studies). The standard quartz electrochemical cell with a volume of 10 mL and a commercially available screen-printed carbon sensor (SPCE, DropSens, Llanera, Spain, Ref. C150) were applied for the experiments. The SPCE sensor consisted of a screen-printed carbon working electrode with a diameter of 4 mm, a silver screen-printed pseudo-reference electrode, and a platinum screen-printed auxiliary electrode.

X-ray photoelectron spectroscopy (XPS) spectra were obtained using a Multi-Chamber Analytical System (Prevac, Rogów, Poland) with monochromated Kα-Al radiation (1486.6 eV) (Gammadata Scienta, Uppsala, Sweden) and an X-ray power of 450 W.

### 3.2. Reagents and Solutions

A 1 mM standard BLM solution was prepared by dissolving an appropriate amount of bleomycin sulfate (Merck, Darmstadt, Germany) in a 0.9% NaCl (saline) solution. This BLM solution was further diluted with saline to obtain BLM solution with a concentration of 10 µM. Sodium acetate and acetic acid used to make an acetate buffer (NaAc—HAc) of pH 4.5, which acted as the supporting electrolyte, were purchased from Merck. There were 1 mM stock solutions of Ni(II), Cd(II), Ca(II), V(V), Fe(III), Mg(II), Cu(II), glucose, ascorbic acid, dopamine, adenine, epinephrine, uric acid and testosterone that were prepared from Merck reagents in deionized water or ethanol (testosterone) and stored at 4 °C in the dark until the influence of interferents was examined. Diethylenetriaminepentaacetic acid (DTPA) was purchased from Merck. Ultra-purified water from a Milli-Q system (Millipore, Livingston, Scotland, UK) was used to prepare the solutions.

### 3.3. Preparation of the pSPCE/PbNPs and Bleomycin (BLM) Analysis

For electrochemical pretreatment and simultaneous decoration of the electrode with nanoparticles of lead (PbNPs), a fresh electrode was placed in a 0.05 M NaAc—HAc solution (pH 4.5) containing 50 µM Pb(II), and subsequently, ten consecutive scans were performed using square-wave voltammetry (SWV). Apart from recording the voltammetric curve, each measurement consisted of electrochemical cleaning at a potential of 0.5 V (E_clean._) for 10 s (t_clean._) and then deposition of PbNPs on the electrode surface at a potential of −1.0 V during 120 s. SWV curves were recorded in the range from −1.0 to −1.7 V using a frequency (f) of 50 Hz, a square-wave amplitude (E_SW_) of 50 mV, and a step potential (ΔE) of 10 mV. After the presented sequence of measurements was performed, the electrode was rinsed with water and allowed to dry in the air. The pretreatment of the electrode was conducted only once before it was used in a series of measurements.

The BLM determination on the pSPCE/PbNPs was performed in the same solution that had been used for the pretreatment of the electrode. Only appropriate amounts of the sample or BLM standard were added to the solution. The square-wave adsorptive stripping voltammetric (SWAdSV) procedure also consisted of the same steps as outlined above, i.e., simultaneous electrodeposition of PbNPs and BLM accumulation at a potential (E_acc._) of −1.0 V for a time (t_acc._) of 120 s, followed by electrochemical cleaning of the electrode surface at a potential of 0.5 V (E_clean._) for 10 s (t_clean._). SWV curves were recorded from −1.0 to −1.7 V with f of 50 Hz, E_SW_ of 50 mV, and ΔE of 10 mV.

### 3.4. Real Sample Analysis

The samples of purified municipal wastewater obtained from a municipal sewage treatment plant (Lublin, Poland), as well as samples of reference material (human urine) (Medidrug Basis-line U), were used for BLM determination at the pSPCE/PbNPs. The samples were spiked with a specific concentration of BLM and analyzed without any preparation.

## 4. Conclusions

In summary, we proposed the application of electrochemical pretreatment (p) of the screen-printed carbon electrode (SPCE) surface and its modification with nanoparticles of lead (PbNPs) in the sample solution as an easy-to-employ method to prepare sensors and further use them for voltammetric determination of the anti-cancer antibiotic bleomycin (BLM). The modification of the electrode with PbNPs is necessary to obtain the BLM signal (no BLM reduction signal was observed at the bare SPCE). Moreover, electrochemical pretreatment does not significantly affect the BLM peak current intensity but causes a slight shift of the peak potential towards less negative potential values and improves the shape of the BLM peak. Furthermore, pretreatment of the sensor before its use in a series of BLM measurements significantly improves the repeatability of the signal.

The results obtained using cyclic voltammetry (CV), electrochemical impedance spectroscopy (EIS), and X-ray photoelectron spectroscopy (XPS) were used to characterize the sensors (bare SPCE, SPCE/PbNPs, and pSPCE/PbNPs). The active surface area (A_s_) increases, and the charge transfer resistance (R_ct_) decreases with PbNPs modification. However, electrochemical pretreatment does not contribute to a change in A_s_ and R_ct_ compared to the SPCE/PbNPs. Most probably, the increased proportion of metallic lead (confirmed by the XPS analysis) affects the repeatability of the deposited lead film before each measurement, thus contributing to the improvement of the shape of the BLM signal and its repeatability.

It was confirmed based on the CV results that the process of BLM reduction on the pSPCE/PbNPs is not purely diffusion- or adsorption-controlled. The developed sensor was validated for selectivity, repeatability, and reproducibility toward BLM. The specific features of the proposed sensor include wide linear ranges (1.0 × 10^−10^–2.0 × 10^−9^ M and 2.0 × 10^−9^–2.0 × 10^−8^ M), very low limits of detection and quantification (2.8 × 10^−11^ and 9.3 × 10^−11^ M, respectively), and a high sensitivity of 0.32 µA/nM. The pSPCE/PbNPs sensor and the SWAdSV procedure were effectively applied for direct analysis of human urine and wastewater samples towards the determination of BLM. The results show the potential of the pSPCE/PbNPs in using it as an electrochemical sensor for direct BLM analysis in real samples with a good recovery rate.

## Figures and Tables

**Table 1:** Characteristics of the sensors.

Electrode	A_S_ [cm^2^]	R_ct_ [Ω cm^2^]	RSD [%] (*n* = 10)	Ref.
SPCE	0.072	146.7	-	[20]
SPCE/PbNPs	0.23	121.5	17.74	This work
pSPCE/PbNPs	0.22	121.3	3.25	[20]

**Table 2:** Comparison of voltammetric analyses of BLM.

Electrode	Method	Linear Range (M)	LOD (M)	Application	Ref.
AuE/DNA	DPV	1.0 × 10^−12^–1.0 × 10^−7^	7.4 × 10^−13^	Serum	[1]
AuE/DNA (E-DNA sensor)	SWV	1.0 × 10^−10^–1.0 × 10^−6^	1.0 × 10^−10^	Serum	[11]
HMDE	AdSV	1.0 × 10^−9^–1.0 × 10^−7^	5.0 × 10^−10^	Serum	[14]
ITO/MB-DNA	DPV	1.0 × 10^−10^–1.0 × 10^−7^	3.3 × 10^−11^	Serum	[15]
pSPCE/PbNPs	SWAdSV	1.0 × 10^−10^–2.0 × 10^−9^ 2.0 × 10^−9^–2.0 × 10^−8^	2.8 × 10^−11^	Urine, wastewater	This work

AuE/DNA—DNA probe modified gold electrode; HMDE—hanging mercury drop electrode; ITO/MB-DNA—methylene blue-DNA modified indium oxide electrode; pSPCE/PbNPs—electrochemically pretreated screen-printed carbon electrode decorated with lead nanoparticles; DPV—differential-pulse voltammetry; SWV—square-wave voltammetry; AdSV—adsorptive stripping voltammetry; SWAdSV—square-wave adsorptive stripping voltammetry.

**Table 3:** The results of BLM determination in reference material of human urine and wastewater purified in a sewage treatment plant.

Sample	BLM Concentration [µM] ± SD (*n* = 3)			Coefficient of Variation * [%]	Recovery ** [%]
	Added	Found SWAdSV	Found in Electrochemical Cell		
Purified wastewater	0.005 0.02	0.0048 ± 0.00012 0.0193 ± 0.00035	0.00048 ± 0.000012 0.00193 ± 0.000035	2.13 1.16	103.5 99.0
RM of human urine	20.0 40.0	20.7 ± 0.44 39.6 ± 0.46	0.00207 ± 0.000044 0.00396 ± 0.000046	2.50 1.81	96.0 96.5

* Coefficient of variation [%] = (SD × 100)/Found SWAdSV, ** Recovery [%] = (Found SWAdSV × 100)/Added.

## Data Availability

The data presented in this study are available on request from the corresponding author.
